# Letter to the Editor: CT Guided Biopsy of a Right Ventricle Primary Cardiac Lymphoma—A Case Report

**DOI:** 10.1007/s00270-023-03482-2

**Published:** 2023-06-19

**Authors:** Thomas J. Vogl, Simon S. Martin, Vitali Koch, Jan-Erik Scholtz, Christian Booz, David M. Leistner, Stephan Fichtlscherer, Teodora Biciusca

**Affiliations:** 1 Department of Diagnostic and Interventional Radiology, University Hospital Frankfurt, 60590 Frankfurt, Germany; 2 Department of Cardiology and Angiology, University Hospital Frankfurt, Frankfurt, Germany

**Keywords:** CT-guided transcutaneous puncture, Primary cardiac lymphoma, Diffuse large B cell lymphoma

Primary cardiac lymphoma (PCL) comprises 1–2% of all cardiac tumors and 0.5% of all lymphomas. Due to its rare occurrence and variety of clinical symptoms, this tumor is difficult to diagnose, and biopsy is indispensable for diagnosis and therapy [1, 2].

We report on a 75-year-old male, immunocompetent patient, who presented with a decrease in general condition and palpitation in the cardiology department of the University Hospital Frankfurt. He complained of thoracic pain and dyspnea on exertion for two weeks. The patient had a history of hypercholesterolemia and gout; however, his family history was negative. Acute coronary syndrome was initially ruled out. Echocardiography revealed inhomogeneous wall thickening in the right ventricular free wall with bulging into the right ventricle, which severely impaired right ventricular function. Subsequent cardiac CT (Fig. 1a) and MRI revealed a large mass along the anterolateral wall of the right ventricle with a diameter of 9.2 cm and a depth of 3.3 cm. Overall, there were no distant metastases or other tumor manifestations. Biopsy was sought for tumor differentiation. Myocardial biopsies obtained by two coronary angiographies via the right femoral vein were inconclusive. Given the percutaneously accessible location of the tumor and the rather broad depth (3.3 cm), a transcutaneous, coaxial CT-guided puncture at the free wall of the right ventricle (Fig. 1b) was finally performed under local anesthesia by using a 18G-puncture needle and 3 samples were taken without any complications. The biopsy was performed after carefully informing the patient about the procedure and risks by an experienced interventionalist performing the procedure (T.J.V.). Immunohistochemically, a diagnosis of DLBCL non-Hodgkin’s Lymphoma was made. The patient’s therapy included recommended chemotherapy. A clear regression of the tumor (8.2 × 1.3 cm) could be seen after therapy, with a residual finding in cardiac CT (Fig. 2) at 10 months after therapy. The remission of symptoms was also achieved.

**Fig. 1:**
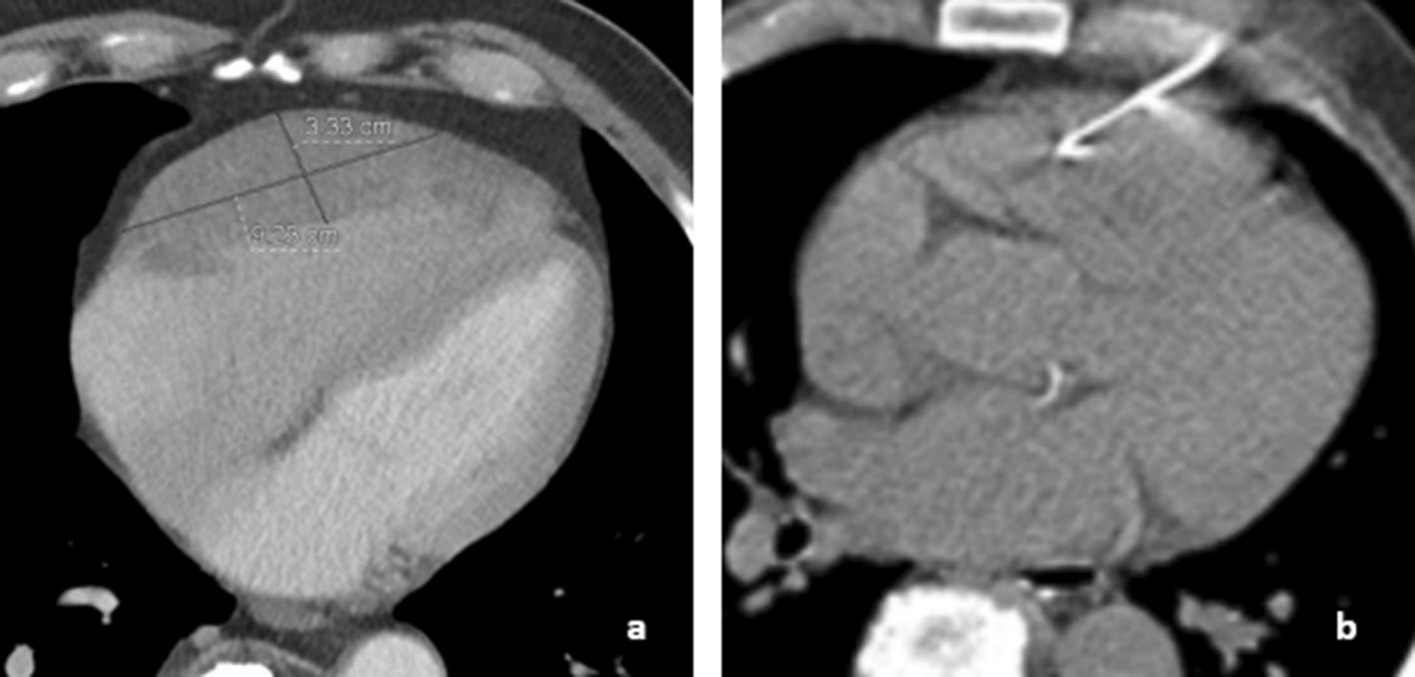
**a** CT Thorax shows a large mass (9.2 × 3.3 cm) along the anterolateral wall of the right ventricle. **b** CT-guided puncture of the free wall of the right ventricle with a 18G-puncture needle

**Fig. 2:**
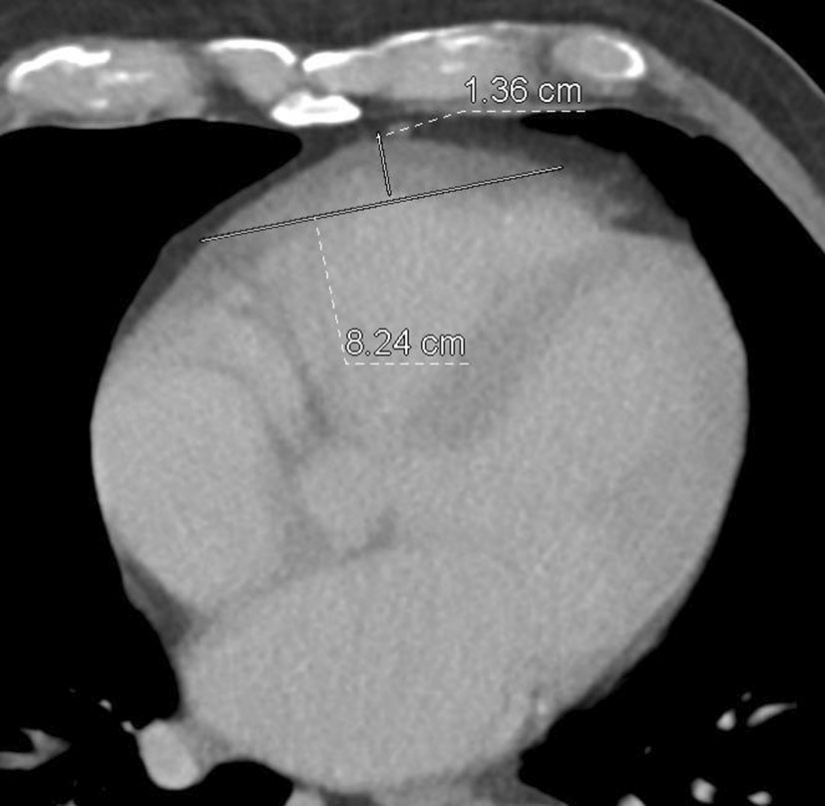
Cardiac CT shows a regredient wall thickening (8.2 × 1.3 cm) of the free wall of the right ventricle compatible with residual findings 10 months after therapy

Being a very rare tumor with non-specific symptoms, the diagnosis of PCL is often made too late, which may lead to a poor prognosis for the affected individuals. Patients are required to undergo several imaging procedures to differentiate PCL from other possible entities, such as angiosarcoma. A biopsy is ultimately mandatory to make the correct diagnosis. [1, 3]. There have been very few reported cases of CT-guided transcutaneous puncture of this kind of tumor due to concerns about the potential complications that can arise from direct puncture of a tumor adjacent to the heart. Serious complications after fine needle aspiration are rare, but there are inherent risks associated with the puncture at the free ventricular wall, such as intrapericardial bleeding or even tamponade and puncture of large vessels. Complications such as pneumothorax or cardiac puncture can be minimized by using CT guidance [4]. In a case report by Yu et al., where a similar case of PCL on the anterior wall of the right ventricle was involved, a biopsy was obtained by transnasal endoscopy from a nasal lymphoma manifestation to make the correct diagnosis [2]. Llibre et al. also reported a less invasive approach in which the examination of a CT-guided fine-needle aspiration cell block enabled an accurate and prompt diagnosis of pericardial angiosarcoma [4]. In our case, a transcutaneous CT-guided puncture of the cardiac tumor was performed without complications, allowing for a quick diagnosis of PCL and therapy initiation.

This case report highlights the high importance of a fast diagnosis of PCL in the prognosis of this rare tumor. Furthermore, it emphasizes that direct transcutaneous CT-guided puncture of the heart is feasible and should be considered a potentially useful tool in diagnosing percutaneous accessible cardiac tumors to facilitate prompt diagnosis and initiation of therapy.

## Declarations

### Human or Animal Participants

All procedures performed in studies involving human participants were in accordance with the ethical standards of the institutional and/or national research committee and with the 1964 Helsinki Declaration and its later amendments or comparable ethical standards.
